# A62 IDENTIFICATION OF SPECIFIC COLONIC DEEP MUSCLE LAYER MACROPHAGES SUBSETS BY THE CD64 (FCΓRI) MARKER

**DOI:** 10.1093/jcag/gwac036.062

**Published:** 2023-03-07

**Authors:** Y Nishihara, S Zarwa, X Bai, G De Palma, S Collins, P Bercik

**Affiliations:** 1 Medicine, McMaster University, Hamilton, Canada; 2 Medicine, Kyushu University, Fuukoka, Japan

## Abstract

**Background:**

Although intestinal muscle layer macrophages have been suggested to play an important role in the colonic transit by interacting with the myenteric plexus neurons, they have not been fully characterized. CD64 (FcγRI) is one of the most generally used markers for intestinal macrophages, but several studies suggested existence of a subpopulation of macrophages that lack CD64. In addition, the muscle layer macrophage subsets currently identified are considered to be same in the small intestine and colon, although this has not been formally tested.

**Purpose:**

In this study, we aim to identify and characterize the subsets of muscle layer macrophages by CD64 marker. We hypothesize that colon specific CD64-macrophages have a different role from CD64+ conventional macrophages.

**Method:**

The muscle layers of small intestine (ileum) and colon were separated from SPF mice and cells from each muscle layer were analyzed by flow cytometry and fluorescent staining. The muscle layer macrophages were gated withCD45+, F4/80+, CD11b+ and Ly6c-, and analyzed with CD64 and MHCⅡmarkers by flow cytometry. In additional experiments, fluorescent staining with CD64 and F4/80 was assessed in whole-mount tissue of the separated muscle layer.

**Result(s):**

Within the macrophage population from the colon muscle layer, we found not only CD64+ cells, a conventional marker of macrophage, but also CD64- cell population (CD45+, F4/80+, CD11b+, Ly6c-). However, in the small intestine, this CD64- cell population was barely detectable. In addition, colonic CD64+ cells had mostly high expression of MHCⅡ marker, while CD64- cells had low expression of MHCⅡ. A similar pattern was found when we examined intestinal and colonic tissues by immunofluorescent staining.

**Conclusion(s):**

We identified a colon-specific CD64- subset of macrophage in muscle layer. Additional experiments are needed to characterize their immunomodulatory properties.

**Please acknowledge all funding agencies by checking the applicable boxes below:**

None

**Disclosure of Interest:**

None Declared

CLIINICAL PRACTICE

